# Vitellogenin-2 acts downstream of PRY-1/Axin to regulate lipids and lifespan in *C. elegans*

**DOI:** 10.17912/micropub.biology.000281

**Published:** 2020-07-21

**Authors:** Avijit Mallick, Bhagwati P Gupta

**Affiliations:** 1 Department of Biology, McMaster University

**Figure 1:**
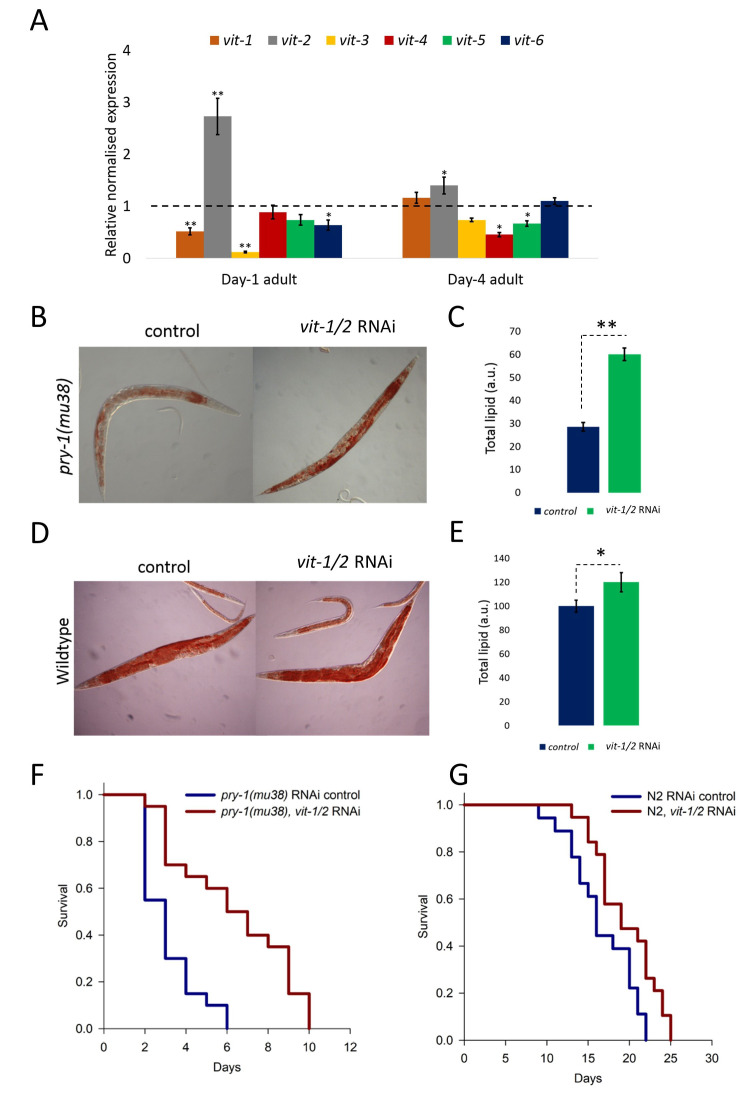
***vit-2* functions downstream of *pry-1* to regulate lipid levels and lifespan. (A)** qRT-PCR analysis of *vit* genes in day-1 and day-4 *pry-1(mu38)* adults. The relative normalized expression has been plotted. Each data point represents the mean of at least two replicates and the error bar represents the SEM. *p<0.05 and **p<0.01, compared to wildtype (N2) which is normalized to one. (**B-E)** Oil Red O analysis of N2 and *pry-1* mutants following control and *vit-1/2* RNAi treatments. B, D) DIC micrographs of representative *pry-1(mu38)* and N2 day-4 adults. (**C, E)** Quantification of Oil Red O signal from two replicates. (**F, G)** Lifespan analysis of *pry-1(mu38)* and N2 animals following adult-specific knockdown of *vit-1/2*. Mean lifespan increased by 102% (6.3 ± 0.6 compared to 3.1 ± 0.3, *p*<0.001) for *pry-1(mu38)* and 16.6%(19.6 ± 0.8 compared to 16.7 ± 0.8, *p*<0.01) for N2*.*

## Description

Lipid metabolism plays an essential role in the survival and adaptation of animals under variable environmental conditions. Lipids are important macromolecules that store energy, serve as structural components, and function as signaling molecules (Watts and Ristow 2017; Papsdorf and Brunet 2019). Defects in lipid metabolism are linked to various diseases and aging in eukaryotes. Therefore, understanding the regulation of this process is critical to modulating disease progression (Wymann and Schneiter 2008).

We have shown earlier that lipid metabolism in *C. elegans* is regulated by an Axin family member, PRY-1 (Ranawade *et al.* 2018). While the signaling network of PRY-1 in this process remains to be investigated, Axin family of proteins are known to function in both WNT dependant and independent pathways to regulate various developmental events (Mallick, *et al.* 2019). Our transcriptomic analysis of both mRNA and miRNA genes revealed that PRY-1 is involved in lipid synthesis by affecting the expression of genes such as fatty acid desaturases (*fat-5, fat-6*, and *fat-7*) and vitellogenins (*vit-1, vit-2, vit-3, vit-4*, *vit-5* and *vit-6*) (Ranawade *et al.* 2018; Mallick, *et al.* 2019).

Vitellogenins are yolk lipoproteins, similar to mammalian apolipoprotein B, that bind to complex lipids and aid in their transportation from the intestine to the gonad (Kimble and Sharrock 1983; Grant and Hirsh 1999). Moreover, *vit-2* has been shown to negatively regulate longevity and such a role of *vit-2* depends on autophagy, lysosomal lipases, DAF-16/FOXO and HLH-30/TFEB (Seah *et al.* 2016). In this study, we report a new, adult-specific role of *vit-2* in *pry-1*-mediated regulation of lipid levels and lifespan. We analyzed the transcript levels of *vit* genes in day-1 and day-4 adults and found that *vit-2* was the only vitellogenin whose expression was significantly upregulated in *pry-1* mutants (Figure 1A). This suggested to us that *vit-2* is negatively regulated by *pry-1* and may be involved in *pry-1*-mediated adult-specific processes. To investigate this further, we examined whether *vit-2* knockdown during adulthood can rescue the lipid and lifespan defect (Mallick, *et al.* 2020) of *pry-1* mutants. This was done using a *vit-1* dsRNA that also knocks down *vit-2* due to the sequence similarity (Ranawade *et al.* 2018). The results showed that the knockdown of *vit-1/2* during adulthood significantly rescued lipid levels in *pry-1(mu38)* (almost 2-fold) (Figures 1B and 1C). Similar experiments in wildtype animals showed a modest increase (by 1.2-fold). We also examined the lifespan phenotype following *vit-1/2* RNAi and observed a marked rescue of the lifespan defect (Mallick *et al.* 2020) in *pry-1(mu38)* (102% increase in mean lifespan). The wildtype animals showed a comparatively lower increase in the mean lifespan (16.6%) (Figures 1F and 1G). Overall, these findings show that *vit-2* functions downstream of *pry-1* to regulate both lipid levels and lifespan.

## Methods

**Strain and growth conditions**

Worms were grown at 20°C on standard nematode growth media plates seeded with *E. coli* OP50. The strains are N2 (wildtype *C. elegans*) and DY220 *pry-1(mu38)* I.

**Lifespan analysis**

Lifespan experiments were performed as described previously (Murphy *et al.* 2003) at 20^o^C. All experiments were performed on RNAi plates with HT115 cells expressing either empty vector (L4440) or dsRNA of *vit-1/2* gene (The Ahringer *C. elegans* RNAi feeding library, sjj_K09F5.2, location X-4A17, FP: CATGCTTGCTTTGTGGAGAA and RP: TTTGAGAATCCTGGGAAACG). Synchronized animals were transferred onto RNAi plates at L4 stage and observed every day throughout the lifespan.

**Oil Red O staining**

Oil Red O staining was performed as previously reported (Ranawade *et al.* 2018). Animals at day-1 adulthood were collected after washing with 1X PBS buffer from the plate and treated as described in the protocol. Animals were mounted and imaged with a Q imaging software and Micropublisher 3.3 RTV color camera outfitted with DIC optics on a Nikon 80i microscope. NIH ImageJ software was used to quantify Oil Red O intensities (Soukas *et al.* 2009). 15 to 30 worms were randomly selected from each category in at least two separate batches.

**qPCR analysis**

Total RNA was extracted from bleach synchronized worms by Tri-reagent (Catalog Number T9424, Sigma-Aldrich Canada) according to the manufacturer’s instructions. Using oligo (dT) primers cDNA was made from total RNA with SensiFAST™ cDNA kit (Catalog Number BIO-65054, USA). Quantitative real-time PCR (qRT-PCR) analysis was performed on a CFX 96 BioRad cycler in triplicate with SensiFAST™ SYBR® Green Kit (Catalog Number BIO-98005, USA), according to the manufacturer’s instructions. Primers used in the study are listed below: *pmp-3* (FP: CTTAGAGTCAAGGGTCGCAGTGGAG and RP: ACTGTATCGGCACCAAGGAAACTGG), *vit-1* (FP: GGTTCGCTTTGACGGATACAC and RP: AACTCGTTGGTGGACTCATC), *vit-2* (FP: GACACCGAGCTCATCCGCCCA and RP: TTCCTTCTCTCCATTGACCT), *vit-3* (FP: GGCTCGTGAGCAAACTGTTG and RP: TTAATAGGCAACGCAGGCGG), *vit-4* (FP: TGTCAACGGACAAGAGGTTG and RP: TCCTTTGGTCCAGAGACCTTC), *vit-5* (FP: GGCAATTTGTTAAGCCACAA and RP: CCTCCTTTGGTCCAGAAACCT) and *vit-6* (FP: AGTCGCTATTGTCGAGCGTC and RP: AGACGGAGGTCACCTTTTGC).

**Statistical analysis**

For lifespan analysis, all statistics were performed using SigmaPlot software 11. Survival curves were estimated using the Kaplan-Meier test, and differences among groups were assessed using the log-rank test. Survival data are expressed relative to the control group. Other statistics were performed using Microsoft Office Excel 365. Bio-Rad CFX manager was used for Ct and *p* values of qPCR analysis.
